# Administration of Simvastatin after Kainic Acid-Induced Status Epilepticus Restrains Chronic Temporal Lobe Epilepsy

**DOI:** 10.1371/journal.pone.0024966

**Published:** 2011-09-19

**Authors:** Chuncheng Xie, Jiahang Sun, Weidong Qiao, Dunyue Lu, Lanlan Wei, Meng Na, Yuanyuan Song, Xiaohua Hou, Zhiguo Lin

**Affiliations:** 1 Department of Neurosurgery, The First Affiliated Hospital of Harbin Medical University, Harbin, China; 2 Department of Neurosurgery, The Second Affiliated Hospital of Harbin Medical University, Harbin, China; 3 Department of Psychiatry, State University of New York Downstate Medical Center, Brooklyn, New York, United States of America; 4 Department of Microbiology, Harbin Medical University, Harbin, Heilongjiang, China; 5 Department of Neurology, The First Affiliated Hospital of Harbin Medical University, Harbin, China; INSERM, UMR-S747, France

## Abstract

In this study, we examined the effect of chronic administration of simvastatin immediately after status epilepticus (SE) on rat brain with temporal lobe epilepsy (TLE). First, we evaluated cytokines expression at 3 days post KA-lesion in hippocampus and found that simvastatin-treatment suppressed lesion-induced expression of interleukin (IL)-1β and tumor necrosis factor-α (TNF-α). Further, we quantified reactive astrocytosis using glial fibrillary acidic protein (GFAP) staining and neuron loss using Nissl staining in hippocampus at 4–6 months after KA-lesion. We found that simvastatin suppressed reactive astrocytosis demonstrated by a significant decrease in GFAP-positive cells, and attenuated loss of pyramidal neurons in CA3 and interneurons in dentate hilar (DH). We next assessed aberrant mossy fiber sprouting (MFS) that is known to contribute to recurrence of spontaneous seizure in epileptic brain. In contrast to the robust MFS observed in saline-treated animals, the extent of MFS was restrained by simvastatin in epileptic rats. Attenuated MFS was related to decreased neuronal loss in CA3 and DH, which is possibly a mechanism underlying decreased hippocampal susceptibility in animal treated with simvastatin. Electronic encephalography (EEG) was recorded during 4 to 6 months after KA-lesion. The frequency of abnormal spikes in rats with simvastatin-treatment decreased significantly compared to the saline group. In summary, simvastatin treatment suppressed cytokines expression and reactive astrocytosis and decreased the frequency of discharges of epileptic brain, which might be due to the inhibition of MFS in DH. Our study suggests that simvastatin administration might be a possible intervention and promising strategy for preventing SE exacerbating to chronic epilepsy.

## Introduction

Epilepsy, characterized by recurrent spontaneous seizures, affects 50 million people worldwide [1]. About 30% of epileptic patients have temporal lobe epilepsy (TLE). TLE, which eventually leads to cognitive deficits, can not be controlled by antiepileptic drugs or surgical removal of epileptic focus [2], [3]. Therefore, there is a need to develop an alternative therapeutic treatment to restrain exacerbation from acute status epilepticus (SE) to chronic epilepsy. TLE is associated with neuropathological changes, including hippocampal sclerosis and neurodegeneration, and extensive reorganization of hippocampal circuits [4]–[8]. Interventions that reduces inflammation cascade and neuron death have been shown to inhibit abnormal spikes and efficacious for restraining TLE after initial precipitating injury (IPI) [9]–[13].

Statins are inhibitors of 3-hydroxy-3-methylglutaryl coenzyme A (HMG-CoA) reductase and inhibit cellular synthesis of cholesterol and isoprenoids [14], [15]. Statins have been recognized for their efficacy in reducing serum cholesterol level and prevention of cardiovascular disease, while growing evidence has shown the efficacy of statins in treating neurodegenerative diseases and possibly traumatic brain injury [16]. The neuroprotective efficacy of statins might be related to their properties such as endothelial protection, antioxidant, anti-platelet effects and anti-inflammatory effects [17]. For instance, statins inhibit a number of inflammatory processes during brain damage and suppress the release of cytokines including interleukin (IL)-1β and tumor necrosis factor-α (TNF-α) in neurological diseases or animal disease models, such as human multiple sclerosis [16]-[18], X-adrenoleukodystrophy [19], experimental autoimmune encephalomyelitis [20], [21], Parkinson's disease [22], spinal cord injury [23], brain ischemia [24], [25], Alzheimer's disease [26], metabolic syndrome [27], and acute brain injury [28]–[30].

A recent study has compared 9 known statins by analyzing parameters possibly related to neuroprotection. Results suggest that simvastatin presents the best characteristics for neuroprotection in neurodegenerative conditions due to its high blood-brain-barrier penetration capacity and cholesterol-lowering effect on neurons [29]. Study from the same group has also suggested that simvastatin is the most effective statin protecting against kainate-induced excitotoxicity and memory impairment [30], which might be related to the role of statins in regulating NMDA receptors [31]. In addition to anti-excitotoxic effect, simvastatin has also shown anti-inflammation effect via reducing expression of interleukin-1 [32]–[35].

Although studies of the efficacy of statins (atorvastatin and simvastatin) in animal model of TLE have shown an anti-seizure effect, only transient outcome (ranging from 3 days to 2 weeks after SE) of statins treatment were evaluated [30], [35]. The long-term effect of simvastatin treatment on seizure development has not been studied. In this study, we evaluated the long-term effect of a 14-day administration of simvastatin on epileptic rat at 4 to 6 months after KA-induced SE.

## Results

### KA-lesion Induced Behavioral Deficits in Rat Model

Within 20–30 min after KA injection (i.c.v), all animals developed status epileptic (SE) and convulsive seizures lasted at least for 1–2 h. Seizure behavior was characterized by animal's rolling toward one side, rotating, and stiffing of limbs and tail. Epileptic rats showed no movement during the interphase of spasms. After a relatively quiescent period (ranging from 10 days to 2 months), 75% rats developed spontaneous seizures that were often evoked by grasping and/or arousing the rats. In the control group, rats with saline injection (i.c.v) did not exhibit any of these abnormal behaviors.

### Simvastatin Down-Regulated the Expression of IL-1β and TNF-α without Altering Expression of IL-6 in the Injured Hippocampus

**Figure 1:**
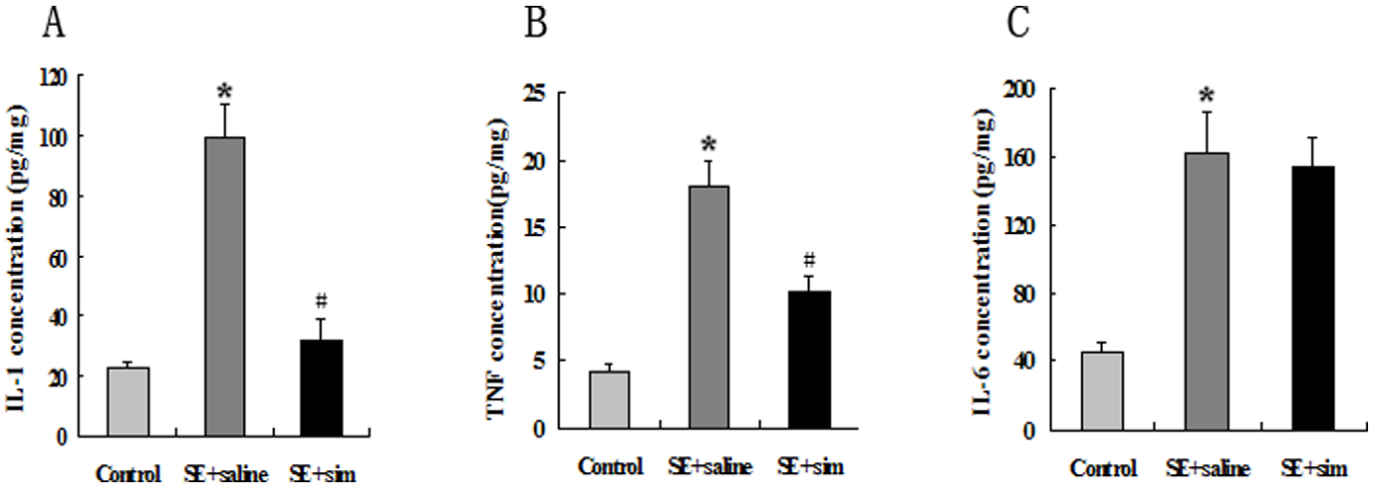
Simvastatin altered the expression level of IL-1β and TNF-α. Bar graphs showed the level of IL-1β (A), TNF-α (B), and IL-6 (C) in the hippocampus 3 days post KA-lesion. KA-injured rats were treated with simvastatin or saline for 3 days. Data were presented as means±standard deviation. *P<0.05 versus control group; #P<0.05 versus the saline group. IL-1β and TNF-α expression was decreased at day 3 after simvastatin treatment compared with that in saline-treated group. However, simvastatin did not suppress the expression of IL-6 compared with the saline-treated group.

We first investigated the effect of simvastatin treatment on the expression of cytokines including IL-1β, TNF-α, and IL-6 in the KA-injured hippocampus. Three days after KA-lesion, significant increases in expression of IL-1β (99.34±11.10 vs 22.12±2.30 pg/mg at normal level), TNF-α (18.10±1.69 vs 4.12±0.68 pg/mg), and IL-6 (163.11±23.45 v.s. 45.12±6.93 pg/mg) were observed in hippocampus (Fig. 1). Compared to saline treatment, 3-day administration of simvastatin significantly suppressed KA-induced up-regulation of IL-1β (31.47±7.10 pg/mg) and TNF-α (10.13±1.19 pg/mg) in hippocampus (Fig. 1A and B), while it showed no effect on lesion-induced up-regulation of IL-6 (154.13±16.97 pg/mg) (Fig. 1C).

### GFAP-positive Astrocytes Reduced after Simvastatin Treatment

**Figure 2:**
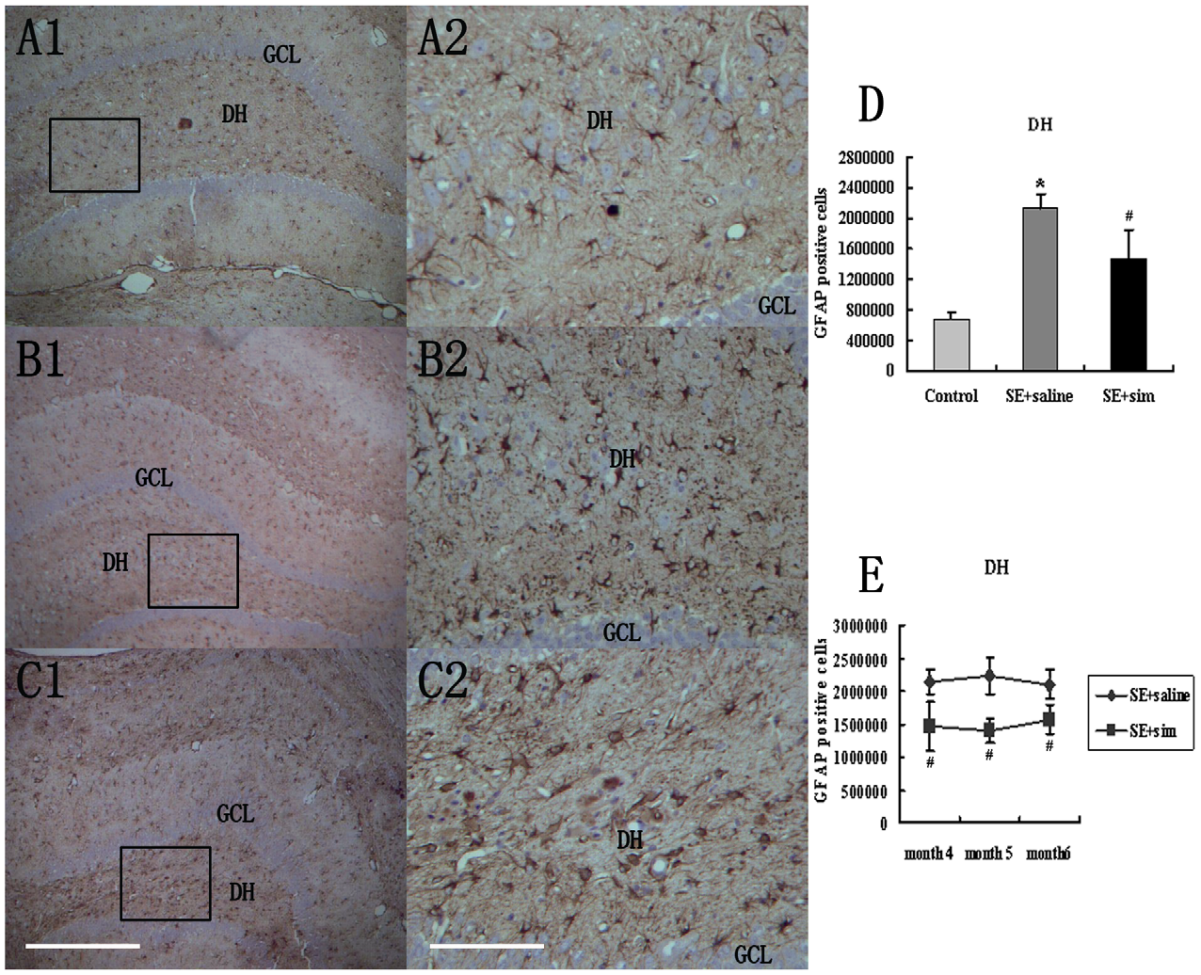
Immunostaining of GFAP for reactive astrocytes in hippocampus at 4, 5 and 6 months post-lesion. (A1–A2) In the normal brain, GFAP staining (brown) showed slim astrocyte morphology and HE staining showed normal neuron (blue) number. Higher magnification of insert was presented in A2. (B1–B2) Simvastatin-treated group exhibited astrocytic hypertrophy and moderate reduction in neurons. (C1–C2) Animals with KA-lesion followed by saline administration showed pronounced up-regulation of GFAP expression. GFAP-positive astrocytes exhibited hypertrophy and atrophied processes. The number of neuron was also severely reduced. (D–E) Quantification of GFAP-positive cells in DH area of hippocampus. Scale bar = 400 µm in C1 (applies to A1, B1, C1); scale bar = 100 µm in C2 (applies to A2, B2, C2). (* P<0.05, vs. the control group; # P<0.05, vs. the saline-treated group).

In response to brain injury, astrocytes become reactive and express various inflammatory mediators that play important roles in the secondary injury. KA-lesion induced reactive astrocytes are often identified by increased immunoreactivity of glial fibrillary acidic protein (GFAP). In the normal brain, only a few astrocytes expressed GFAP and GFAP-positive astrocytes showed slim morphology as well as thin and long processes (Fig. 2A1 and A2). Two-week simvastatin treatment attenuated this lesion-induced GFAP immunoreactivity at 4 months in the epileptic rats. GFAP-positive astrocytes also lost fine processes (Fig. 2B1 and B2). In contrast, 4 months after KA-lesion, saline-treated group showed a robust increase in GFAP immunoreactivity and hypertrophic morphology of astrocytes. (Fig. 2C1 and C2). Quantitative data showed that the density of GFAP-positive cells in dentate hilar (DH) area was reduced compared to saline-treated epileptic rats (Fig. 2D). We also evaluated the reactive astrocytosis at 5 and 6 month post KA-lesion. Our data showed that the simvastatin-mediated suppression in reactive astrocytosis lasted until 6 month post-lesion (Fig. 2E).

### Simvastatin Administration Restrained KA-induced injury in Hippocampal Cytoarchitecture

**Figure 3:**
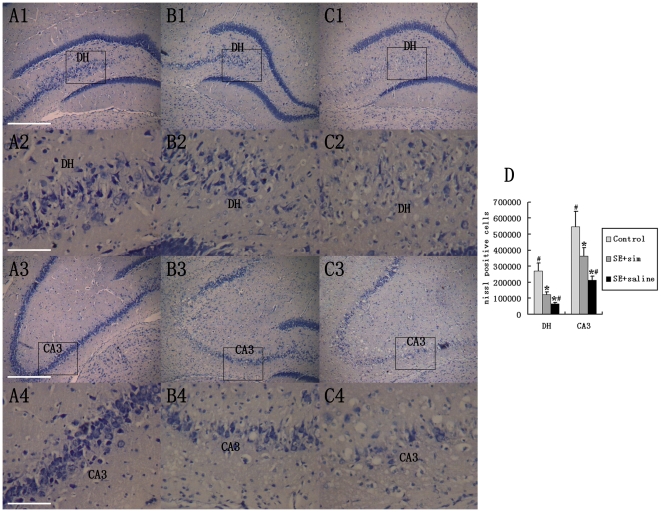
Hippocampal cytoarchitecture visualized with Nissl staining. A1, A2, A3 and A4 showed hippocampal regions from rats with intracerebroventricular saline injection. B1, B2, B3 and B4 showed regions of hippocampus from KA-injured rat followed by simvastatin treatment. C1, C2, C3 and C4 showed hippocampal regions from KA-injured rat followed by saline-treatment. D showed quantitative data of the number of neurons in DH and CA3. scale bar = 400 µm in A1 (applies to B1, C1, A3, B3, C3); scale bar = 100 µm in A2 (applies to B2, C2, A4, B4, C4 ). (* P<0.05, vs. control group, # P<0.05, vs simvastatin-treated group).

Hippocampal cytoarchitecture was evaluated using Nissl staining in different groups at 4 months after the KA-lesion. In this study, the number of neurons in the CA3 and DH region was counted and compared among each group (Fig. 3). Out results showed that KA-lesion induced dramatic degeneration of neurons in CA3 and DH in ipsilateral hippocampus at 4 months. However, the number of neurons in the simvastatin-treated group was greater than that of the saline-treated group, which persisted over time. These data suggest that simvastatin treatment can rescue the loss of neurons in CA3 and DH region.

### Simvastatin Treatment Restrains KA-lesion Induced Mossy Fiber Sprouting (MFS)

**Figure 4:**
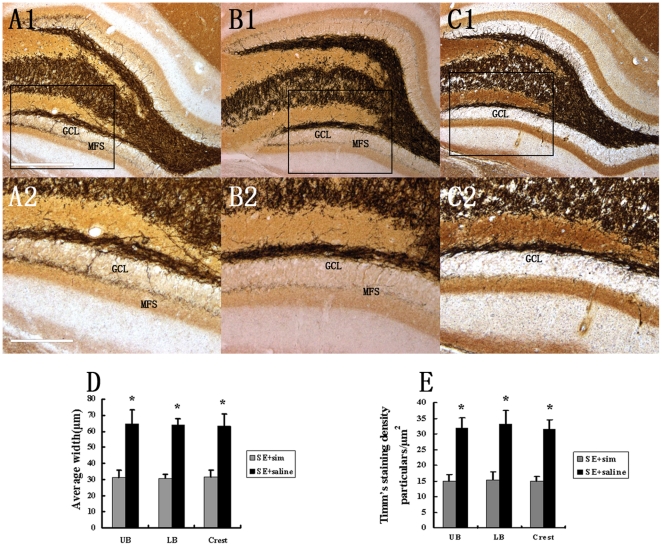
Comparison of KA-lesion induced MFS using Timm's staining in DH. The extent of aberrant MFS in saline-treated group with severe hippocampal injury (A1and A2), simvastatin-treated group with moderate hippocampal injury (B1 and B2) and in comparison with rats with intracerebroventricular saline injection (C1 and C2), visualized by Timm's histochemical staining. Quantitative data for width and density of sprouting into DSGL were presented in (D and E). GCL, granule cell layer, UB, upper blade; LB, lower blade. Scale bars = 500 µm in A1 and 200 µm in A2. (*P<0.05, vs. the simvastatin-treated group).

Mossy fibers from granule cells in the DH undergo reorganization of their terminal projections in both human epilepsy and animal models of epilepsy. In this study, KA lesion-induced aberrant MFS was shown by Timm's method that selectively labeled synaptic terminals of mossy fibers due to their high zinc content. The effect of saline and simvastatin-treatment on KA-induced MFS was assessed at 6 months post-lesion. In the saline-treated group, Timm's staining showed robust MFS that extended into the dentate supragranular layer (DSGL) (Fig. 4A1, A2). On the contrary, in the simvastatin-treated group (Fig. 4B1, B2), supragranular MFS was less intense and more dispersed, though it is still more dense than that in the normal brain (Fig. 4C1, C2). We further compared the average width and Timm's staining density between simvastatin- and saline-treated groups. We found that simvastatin-treatment reduced MFS width and staining density in all upper blade (UB), lower blade (LB) and crest area of DH. These data suggest that simvastatin-treatment restrained KA-induced abberant MFS.

### Simvastatin Inhibits Abnormal Spike Discharges of the Epileptic Brain as Observed by EEG Monitoring

**Figure 5:**
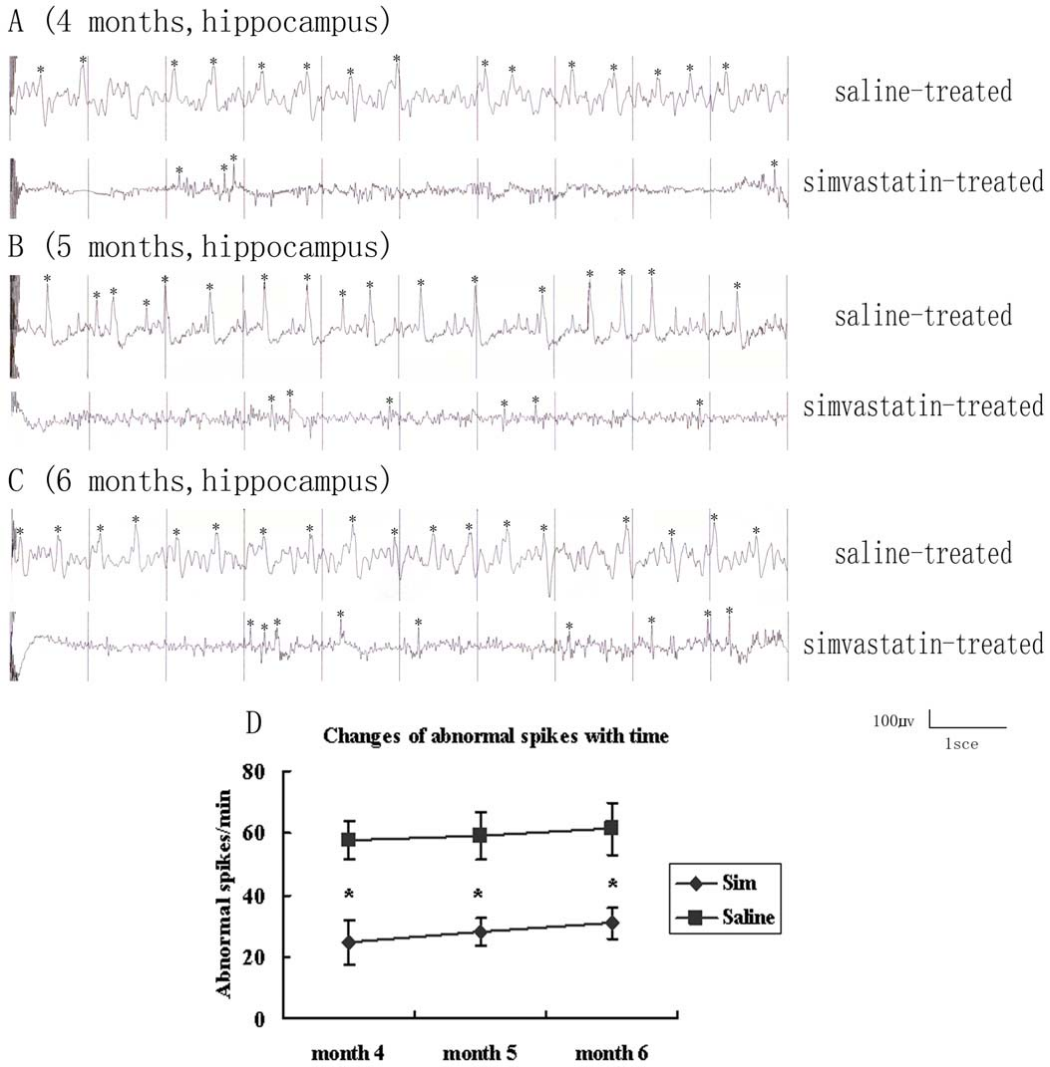
Simvastatin-treatment decreased the frequency of abnormal spikes of epileptic brain. Representative EEG recordings from saline-treated group and simvastatin-treated group at 4 months (A), 5 months (B) and 6 months (C). Quantitative data was presented in (D). *P<0.05, vs. the saline-treated group.

The activity from the hippocampus was monitored with electronic encephalography (EEG) during 4 to 6 months after KA-lesion. Abnormal spikes in EEG showed high amplitude and sharp peak and they were manifested by asterisks (Fig. 5A–C). The EEG data from hippocampus of rats consisted of waves with a frequency ranging from 0 to 75 Hz and amplitude of 500 µV. Quantitative data showed the comparison of EEG data at 4, 5, and 6 months after KA-lesions. Simvastatin-treated group presented a significant lower frequency (4 months: 24.8±7.2/min, 5 months: 28.1±4.2/min, 6 months: 31.0±5.1/min) of abnormal spikes than the saline-treated group (4 months: 57.8±6.4/min, 5 months: 59.34±7.8/min, and 6 months: 61.4±8.6/min). These results suggest that simvastatin-treatment in injured hippocampus could reduce the abnormal discharge of the epileptic brain.

### Statin attenuates seizure behavior induced by kainic acid

**Figure 6:**
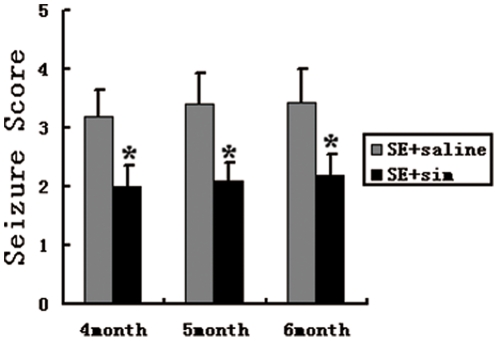
Simvastatin attenuated KA-induced seizure behavior in rats. The rats were orally treated with simvastatin (1 mg/kg) for 0.5 h after KA injection (1.0 µl of KA. i.c.v). The seizure behavior test (seizure score) was performed during 4–6 months after KA injection (1.0 µl of KA. i.c.v). Simvastatin attenuated the seizure score. Data were presented as means±standard deviation. *P<0.05 versus the saline group.

In the present study, we observed seizure behavior (seizure score) at 4-6 months after KA injection (i.c.v) (Fig. 6). The rats were orally treated 0.5 h with simvastatin (1 mg/kg) after KA injection (1.0 µl of KA. i.c.v). Simvastatin attenuated the seizure score. Seizure activity was rated according to the Racine's scale: Stage I, facial clonus; Stage II, nodding and wet dog shaking; Stage III, unilateral forelimb clonus with lordotic posture; Stage IV, lateral forelimb clonus with rearing; Stage V, bilateral forelimb clonus with rearing, jumping, and falling.

## Discussion

In this study, we found that 14-day simvastatin-treatment immediately after KA-lesion was effective in long-term inhibition of TLE until 6 months, which might be due to the suppression of reactive astrogliosis and neuroinflammation. Simvastatin treatment also attenuated aberrant MFS from granular cells in DH, which may lead to decreased discharges of epileptic brain. These findings suggest that simvastatin could reduce KA-induced lesion in hippocampus and simvastatin administration may become a possible intervention for impeding the progression of status epilepticus to chronic epilepsy.

### Simvastatin inhibits neuroinflammation and reduces reactive astrocytosis

Pro-inflammatory cytokines play key roles in the epileptogenic cascade [9]–[13], [36]–[38] including seizure-related pathological changes in hippocampus, such as neuronal death, reactive gliosis and abberant mossy fiber sprouting. In rat model of KA-induced epilepsy, injury in hippocampus occurs accompanied by upregulation of cytokines including TNF-α and IL-1 in glial cells. Production of cytokines further precedes neuronal degeneration [39], [40]. Reducing TNF-α level by employing thalidomide has showed effective antiepileptic activity [41]. IL-1β, another critical cytokine involved in epileptogenesis, has been reported to increase seizure susceptibility in rats brain [42]. Intracerebral injection of high dose IL-1β, which is sufficient to generate limbic seizures in wild type mice, fails to cause seizures in transgenic mice with deficient IL-1β receptors [43]. The effect of IL-6 in epileptic brain is still controversial. Some studies indicate that IL-6 might be neuroprotective through activating the synthesis of corticotrophin and glucocorticoids, and initiating an anti-inflammatory feedback loop [44], [45]. Therefore, manipulating level of cytokines in inflammatory cascades could have a potential effect on KA-induced injury.

Previous studies suggest a role of simvastatin in suppression of cytokines by regulating nuclear factor-κB transcription pathway [46] and decreasing isoprenylation protein involved in cellular signaling [47] or Rac1/PI3K/PKB-dependent caspase-1 activation [48]. In this study, we observed simvastatin-induced decrease in IL-1 β and TNF-α in injured hippocampus as early as 3 days after KA-lesion. The reduction of IL-1 β and TNF-α in early stage after injury attenuates secondary inflammation damage, which may contribute to the improvement of pathological changes in hippocampus. Our founding supports the hypothesis that simvastatin-mediated early suppression of the inflammatory cascade may result in a marked reduction in secondary neuronal damage after SE.

Evidence has also shown that simvastatin-treatment increases brain-derived neurotrophic factor (BDNF)expression in hippocampus after traumatic brain injury (TBI) [34]. Moreover, supplementation of BDNF attenuates astrocytosis and IL-1β expression after status epilepticus [49]. Therefore, the neuroprotective effect of simvastatin observed in this study may be also related to increased BDNF level that prevents neurons from lesion-induced degeneration. Thus, both anti-inflammation and neurotrophy effect of simvastatin may contribute to its neuroprotective role found in our study.

Astrocytes also play a critical role in epileptogenesis [50]–[52], particularly in chronic relapsing forms, which is associated with the change of astrocytic potassium and calcium-signaling. Reactive astrocytosis is also a source of cytokines including IL-1β, TNF-α, and IL-6 [53]. Reactive astrocytosis may exacerbate inflammation by inducing the migration of other leukocytes into the injured site, interrupting blood-brain-barrier function [54], [55], and producing reactive oxygen species[56], [57] and cytotoxic edema [58]. Therefore, we also investigated the effect of simvastatin-treatment on the extent of reactive astrocytosis after KA-lesion. Our result showed that simvastatin-treatment reduced GFAP-positive astrocytes in both DH and CA3 area in the hippocampus where neuron loss mainly occurred. This simvastatin-mediated suppression of astrocytosis may contribute to inhibition of neuroinflammation and neuronal loss, thus exert a neuroprotective role in KA-induced injury.

### Simvastatin attenuates aberrant MFS into the DSGL

In both human and animal models of epilepsy, due to the degeneration of pyramidal neurons in CA3 and interneurons in DH, axons of the granular cells (mossy fiber) lose their normal connecting targets and sprout abnormally, which is called aberrant MFS. The extent of MFS is influenced by the degree of neuron loss in CA3 and DH. [59]–[61]. This is supported by observation that intense MFS occurred associated with extensive loss of CA3 pyramidal neurons and hilar neurons in not only animal models of TLE but also TLE patients. [61], [62]–[64]. Therefore, decreased neuronal death after simvastatin-treatment in injured hippocampus may contribute to reducing MFS.

Aberrant sprouting of mossy fiber into DSGL in KA-injured hippocampus is known to contribute to spontaneous seizures [62]–[65]. The extent of MFS has been correlated with increased seizure susceptibility of spontaneous seizures [64], [66], [67]. Therefore, we speculate that a lasting suppression of aberrant MFS is likely to reduce the recurrent excitation and seizure susceptibility. In this study, we showed that KA-injured animals with 2-week administration of simvastatin presented a significant decrease in the frequency of discharge in 4–6 month after KA-lesions, which was concurrent with restrained MFS in DG. This observation suggests that simvastatin-treatment could reduce the abnormal discharge of the epileptic brain possibly through restraining the excitation connection derived from aberrant MFS. In addition to MFS, high frequency of spontaneous recurrent motor seizures (SRMS) is also associated with other features of chronic epilepsy, such as extensive loss of inhibitory interneurons in hilar area [68], [69] and changes in neurotransmitters expression by granule cells [70]. In the future study, it will be intriguing to assess the loss of inhibitory interneurons and neurotransmitter expression after simvastatin treatments.

### Possible mechanism underlying anti-excitotoxic effect of simvastatin

The possible mechanisms underlying the beneficial effect of statins on neurodegenerative and cardiovascular diseases have been extensively investigated and discussed in previous literature. While the study of the effect of statins in epileptic conditions has been limited, previous research has shown the evidence supporting the anti-excitotoxic effect of statins [17], which might explain the beneficial effect of simvastin after KA-induced injury. For instant, in vitro study has shown that statins, especially rosuvastatin and simvastatin, protect cultured neurons from NDMA-induced excitotoxic death [31].

Atorvastatin has also been shown to protect primary cortical neurons from glutamate-induced excitotoxicity possibly by attenuating glutamate-induced intracellular calcium signaling that modulating NMDA receptor function [71]. Anti-excitotoxic and anti-seizure effects of statins have been further studied in KA model of TLE. Pre-treatment with atorvastatin efficiently reduced KA-induced seizure activities and neuronal loss [35]. Moreover, a very recent in vivo study, which compared 5 commercially available statins in KA model of TLE, suggests that simvastatin is the most effective statin in attenuating KA-induced excitotoxicity and improving memory [30]. Our results in the present study further support the anti-excitotoxicity effect of statins in epileptic conditions and this protective effect could last until 6 months after KA-induced SE. Considering previous studies together with our results, we suggest that the roles of statins in regulating glutamate receptor and inhibiting injury-induced inflammatory response may contribut to the beneficial effect in the epileptic conditions.

### Simvastatin shows a long-term neuroprotective effect on epileptic brain

Previous studies have investigated effects of two statins, atorovastatin [35], [46], [72] and lovastatin [73], [74], on neuronal protection during epileptogenesis and attenuating seizure behavior in various animal models, including pentylentetrazole-kindled rats, quinolinic acid-induced seizure, pilocarpine-induced status epileptic and kainic-acid induced seizure. A very recent study has compared 9 statins by analyzing several parameters that could be related to neuroprotection and suggests that simvastatin presents the best characteristics for preventing neurodegenerative conditions, due to its high BBB penetration capacity and cholesterol lowering effect on neurons [29]. Moreover, study performed by the same group also shows that simvastatin is the most efficient statin in protecting against KA-induced excitotoxicity and memory deficits [30]. However, all animal studies above have mainly focused on the transient effect of the statin administration, ranging from 3 days to 2 weeks after SE. In the present study, we evaluated the long-term neuroprotection of simvastatin at 4, 5 and 6 months after KA-induced SE and our data showed a consistent result as transient outcome, suggesting that a 14-day administration of simvastatin has a beneficial effect that can last for 6 months. Our data also support previous studies of transient outcome of statins administration. This long-term effect of simvastamin suggests that it may have therapeutic potential in the treatment of neurodegenerative disease involving excitotoxicity.

### Conclusion

Our data showed that 2-week administration of simvastatin immediately after KA-induced seizure lead to reduced astrocytosis, attenuated neuronal loss, decreased abnormal MFS and reduced seizure activity in the brain at 4 to 6 month after KA lesion, suggesting a long-term protective role of simvastatin in the epileptic brain. This study supports the possibility of using simvastatin administration as a neuroprotective intervention for retarding progression of the epileptogenesis and a therapeutic strategy for epilepsy.

## Materials and Methods

### Experimental Groups

Adult male Wister rats (200–250 g) were used in our experiment and all animals were maintained under 12 h light/dark-cycle with free access to food and water in a temperature-controlled room. All animal procedures were in accordance to guidelines of Ethics Committee of the First Clinical College of Harbin Medical University (201001). Rats were divided into 3 groups: 1) SE were induced in rats with KA injection followed by oral administration of saline starting at 0.5 h after SE once a day for 14 consecutive days; 2) Rats were subjected to KA-lesion followed by oral administration of simvastatin (1 mg/kg/day) starting at 0.5 h after SE for 14 consecutive days. The dose was selected according to our previous study [75]; 3) Rats were subjected to intracerebroventricular saline injection as a control. Rats with KA-lesion were sacrificed at 3 days, 4 and 6 months after simvastatin- or saline-administration. Rat cerebral hemispheres were homogenized for enzyme-linked immunosorbent assay (ELISA) at 3 day after KA-lesion.

### Kainic acid lesion

Rats were anesthetized with chloral hydrate (40 mg/kg body weight, i.p.), then immobilized in a stereotaxic apparatus (WPI Stoelting, USA) with a plane of incision bar set at 3.3±0.33 mm below the interaural line. After making a midline incision, the skull was exposed and one burr hole was drilled according to the coordinates: 3.7 mm posterior to the bregma, 4.1 mm lateral to the midline, and 3.5 mm under the dura. Each rat was injected with 1.0 µl of KA (0.5 µg in 1.0 µl Saline) in the right lateral ventricle at the speed of 0.2 µl /min. The epileptic rats were further used for experiments.

### Tissue processing

Rats were deeply anesthetized with chloral hydrate (40 mg/kg body weight, i.p.). For Timm's staining rats were perfused with 100–150 ml sodium sulfide followed by post-fixation in 4% paraformaldehyde (PFA) for 10 h and then brains were transferred into cryo-protection solution (30% sucrose). For Nissl staining, HE staining and immunohistochemistry rats were perfused with phosphate-buffered saline (PBS) followed by post-fixation in 4% PFA for 72 h and brains were embedded with paraffin.

### Nissl staining

The surviving neurons in the hippocampus were visualized by Nissl staining [76], [77]. The mounted sections were rehydrated in distilled water, and submerged in 0.5% cresyl violet solution for 10 min until the desired depth of staining was achieved.

### GFAP staining

After deparaffinization, brain sections were incubated in 2% bovine serum albumin (BSA) in PBS at room temperature for 30 min, and subsequently incubated with mouse anti-GFAP antibody (1∶160 diluted in PBS; sigma) at 4°C overnight. After washing in PBS, sections were incubated in peroxidase blocking solution for 10 min at room temperature. Sections were then sequentially incubated with biotin-conjugated anti-mouse immunoglobulin G (1∶100 dilution; sigma) for 10 minutes, streptavidin-HRP in PBS for 30 min, and DAB solution for 1-3 min. Sections were washed in PBS between each step and all these incubation were performed at room temperature. Sections were then counterstained with HE, rinsed in distilled water, dehydrated through 95% ethanol for 2 min, 100% ethanol for 2×3 min and then cleared in xylene for 2×5 min for coverslip mounting.

### Timm's staining

Serial coronal sections (8 µm) were collected with a cryocut (Leica VT 1000S). Every fifth section was collected on slides and dried at room temperature overnight. For the visualization of MFS, sections were developed using the following solution: 120 ml of 50% gum arabic, 10 ml of 47% sodium citrate buffer, 10 ml of 51% citrate buffer, 60 ml of 5.78% hydroquinone and 212.25 mg silver nitrate. The physical development was performed in the dark at room temperature for 120 min. The sections were rinsed in tap water, then dehydrated, cleared, and coverslipped in Entellan (107961.0100; Merck). Evaluation of Timm's staining was performed according to the method introduced by Shetty and Turner [78]. Both the average width and the density of the MFS in the DSGL were measured in all regions of the DG (The upper and lower dentate blades and the crest). The scores were recorded for the DG in coronal sections from the ipsilateral dorsal hippocampus by an observer who was blind to the groups.

### ELISA

ELISA kit (Promega Corp. Madison, WI) was used to determine the protein concentration and the procedure was performed according to the instructions provided by the company. Lysis buffer was added to the tissue sample and the protein was obtained by centrifuging the solution at 14000 rpm for 12 min at 4°C. The expression of IL-1β, TNF-α, and IL-6 at 3 days after SE were measured using equal amounts of lysate from hippocampus tissues.

### Electrode implantation

At 4 months after KA-lesion, the rats were again anesthetized with chloral hydrate (40 mg/kg body weight, i.p.) and fixed into stereotaxic apparatus (WPI, Stoelting, USA). A bipolar electrode, consisting of two twisted strands of stainless steel wire, was stereotactically implanted into the right hippocampus using the following coordinates: AP = −5.6 mm, L = 4.5 mm and V = 2.6 mm. A single stainless steel wire was inserted into the left frontal cortex acting as earth electrode. The electrodes were attached to male connector pins, which were inserted into a connector strip. Stainless steel screws were threaded into the right and left parietal cranium to help fix the electrode. The electrode assembly was then fixed to the skull with acrylic resin before the scalp was sutured.

### EEG examination and analysis

Starting at 3 days after electrode implantation, all rats underwent 3 EEG examination sessions per week with 4 h of continuous monitoring per session. Recordings were performed in the afternoon to minimize potential circadian variations, following the habituation of the rats to the test cage. For spike evaluation, the EEG from the hippocampus of rats consisted of waves with a frequency ranging from 0 to 75 Hz. The data were collected to evaluate average spikes at 4 to 6 months post KA-lesion.

### Image analysis

Imaging analysis was performed according to method describe previously [79]. A series of every fifth section of each animal were used for analysis. For neuron counting, in each section, the contour of CA3 pyramidal cell layers, and DH was first delineated with the tracing function of StereoInvestigator(Microbrightfield Inc.,Williston, VT). After this, the optical fractionator component was activated, and the number and location of the counting frames and the counting depth for that section were determined by entering parameters such as the grid size (200×200 µm for CA3 cell layers, and 60×60 µm for the DG), the thickness of the guard zone (4 µm), and the optical dissector height (8 µm). A computer-driven motorized stage hen allowed the section to be analyzed at each of the counting frame locations. All nissl-positive cells that were present in the 8 µm section depths were counted in each of the chosen serial sections. The StereoInvestigator program calculated, based on the above-described parameters and cell counts, the total number nissl-positive cells in each of the above-mentioned hippocampal regions. For GFAP counting, similar method was used and the total number of GFAP-positive astrocytes was quantified in DH and CA3 area.

### Statistical analysis

All data were presented as mean±SEM. One-way analysis of variance (ANOVA) with Newman-Keuls multiple comparisons test was employed for comparison. The operations were performed using StatView software (version5.0, SASinstitute Inc., Cary, NC, USA). Statistical significance was set at P<0.05.
